# Prognostic and Clinicopathological Significance of Long Non-coding RNA PANDAR Expression in Cancer Patients: A Meta-Analysis

**DOI:** 10.3389/fonc.2019.01337

**Published:** 2019-12-03

**Authors:** Lizhi Han, Bo Wang, Ruoyu Wang, Zijian Wang, Song Gong, Guo Chen, Dionne Telemacque, Yong Feng, Weihua Xu

**Affiliations:** ^1^Department of Orthopaedics, Union Hospital, Tongji Medical College, Huazhong University of Science and Technology, Wuhan, China; ^2^Department of Rehabilitation, Wuhan No.1 Hospital, Wuhan Hospital of Traditional Chinese and Western Medicine, Wuhan, China; ^3^Department of Orthopedics, Xiangyang Central Hospital Affiliated Hubei University of Arts and Science, Xiangyang, China

**Keywords:** long non-coding RNA (lncRNA), meta-analysis, cancer, promoter of CDKN1A antisense DNA damage activated RNA (PANDAR), prognosis

## Abstract

**Background:** Long non-coding RNA PANDAR is an emerging non-coding RNA mapping to 6p21.2. It underlies metastatic progression and chromosomal instability in a variety of cancers. Despite the fact that recent studies have revealed that lncRNA PANDAR may be a potential prognostic biomarker for patients with cancer, there has still been controversy on the prognostic value of PANDAR.

**Methods:** Databases of PubMed, Embase, SinoMed, and Web of Science were carefully searched and the literature which investigated the prognostic value of PANDAR expression among human cancers was collected for further analysis. Odds ratios (ORs) or hazards ratios (HRs) with 95% confidence intervals (CIs) were pooled to estimate the relation between PANDAR expression and survival or clinicopathological characteristics of cancer patients.

**Results:** There were 13 eligible studies in total, with 1,465 patients enlisted in this meta-analysis. All the eligible studies complied with the case-control study. The outcome showed that the elevated expression level of PANDAR was significantly related to poor overall survival (OS) (pooled HR 1.72, 95%CI 1.14–2.60). However, high or low expression of PANDAR did not differ in the prediction of event-free survival (EFS). Moreover, we discovered that high PANDAR expression was closely related to decreased OS in colorectal cancer (pooled HR 3.43, 95%CI 2.06–5.72) and reduced expression level of PANDAR was markedly related to poor OS (pooled HR 0.65, 95%CI 0.45–0.88) in non-small cell lung cancer. However, the expression level of PANDAR had no significant association with OS in renal cell carcinoma (pooled HR 1.19, 95%CI 0.56–2.50). Moreover, after analysis, we discovered that the high expression level of PANDAR was associated closely with the depth of invasion (pooled OR 3.95, 95%CI 2.36–6.63), lymph node metastasis (pooled OR 1.92, 95%CI 0.93–3.98), tumor stage (pooled OR 2.05, 95%CI 0.99–4.27), and distant metastasis (pooled OR 2.87, 95%CI 1.60–5.16).

**Conclusions:** Our study revealed that increased PANDAR expression may serve as an adverse prognostic biomarker for cancer patients, thus helping the clinical decision-making process.

## Introduction

Despite the remarkable advances in the management and treatment of cancer patients over the years, cancer continues to be a major public health issue and there are many problems remaining to be solved (1, 2). It is estimated that new cases of cancer will be as many as 18.1 million while cancer deaths worldwide will be up to 9.6 million in 2018 (3). As new cases and deaths from cancer increase annually, it is expected that the burden of cancer will aggravate in tandem, especially in less developed countries (4). Since the diagnosis of various cancers is confirmed at advanced stages rather than the early stages, the prognosis of cancer is poor. Therefore, identification and validation of novel applicable cancer biomarkers with high sensitivity and specificity are crucial for predicting prognosis and performing targeted therapy (5). Clinicians may use some potential prognostic biomarkers to make an early diagnosis and choose the optimal therapeutic schedule.

The long non-coding RNAs (lncRNAs) are a new category of non-coding RNAs measuring more than 200 nucleotides in length, lacking the ability to encode any protein (6). The genome-wide studies have shown that the majority of the human genome are dynamically transcribed to create a large portion of lncRNAs (7). Increasing evidence has been provided by the application of next-generation sequencing technologies for lncRNA dysregulation in cancer. Accumulating evidence indicates that lncRNAs exert synergetic functions on tumorigenesis or tumor suppression, and abnormal expression of lncRNAs may respond to cell proliferation, tumor progression or metastasis (8, 9). LncRNAs are functionally categorized into two major categories of tumor suppressors and oncogenes (10). As shown from previous studies, lncRNAs might act as transcriptional regulators, splicing modulators, enhancers, post-transcriptional processors, scaffolds, or molecular decoys by interacting physically with proteins or other types of RNA, thereby directly affecting the cellular signaling cascades (11, 12). Functional lncRNAs were identified as promising biomarkers for diagnosing cancer and predicting tumor prognosis, and could also be utilized as potential therapeutic targets (13).

PANDAR (promoter of CDKN1A antisense DNA damage activated RNA), initially reported by Hung et al. is an emerging noncoding RNA with 1,506 nucleotides in length mapping to chromosome 6p21.2 (14). As discovered by Hung et al. PANDAR is induced after DNA damage in a p53-dependent pattern, limiting the expression of pro-apoptotic genes in fibroblasts by interacting with the transcription factor NF-YA (14). Recently, PANDAR was known as biomarkers of cancer and potentially involved in the instability of chromosomes and cancer metastatic progression (15, 16). It was found in previous studies that PANDAR was upregulated in various cancers, including gastric cancer (17), cholangiocarcinoma (18), hepatocellular carcinoma (19), and clear cell renal cell carcinoma (20). On the other hand, it was downregulated with poor prognosis in non-small cell lung cancer (21). The obvious tissue-specific expression patterns of lncRNA relative to protein-coding genes may result in the inconsistent expression of PANDAR in cancer (22, 23). In conclusion, there is still controversy about the prognostic value of PANDAR in cancer patients due to the distinct outcome and limited sample size in most studies reported to date. Hence, we conducted a current and comprehensive meta-analysis to elucidate the prognosis and clinicopathological significance of PANDAR expression in patients diagnosed as cancer.

## Materials and Methods

### Study Strategy

All procedures mentioned below were performed in accordance with PRISMA Checklist and Cochrane Collaboration protocols (24, 25). Two researchers (Lizhi Han and Bo Wang) searched the databases PubMed, SinoMed, Embase, and Web of Science independently to collect all articles associated with the prognostic value of aberrantly expressed PANDAR in malignancy patients. The literature search was completed on November 11, 2018. The detailed example of the full electronic search strategy for PubMed is provided in Supplementary Material 1. In order to heighten the sensitivity of the search, both free-text words and MeSH terminology were utilized in the search strategy. The search strategy included: “PANDAR or long non-coding RNA PANDAR, human” AND “tumor or cancer or neoplasm or carcinoma or malignancy” AND “prognosis or prognostic or outcome or survival.” The references of articles collected for our study were screened to obtain the eligible literature. There were discussions among the groups to settle any conflicts.

### Inclusion and Exclusion Criteria

Studies that complied with the following criteria were eventually included: (1) Study design: case-control study; (2) Population: patients were pathologically diagnosed with any type of human malignancy; (3) Intervention and Comparison: patients were divided into negative and positive expression or low and high expression group according to the expression levels of PANDAR, the patients whose expression levels of PANDAR are positive or high belong to intervention group while the patients whose expression levels of PANDAR are negative or low belong to comparison group. Any applicable techniques were used to measure the expression level of lncRNA-PANDAR in human tissues; (4) Outcomes: the connection between PANDAR expression level and survival outcome was examined including overall survival and event-free survival; (5) Sufficient published data or the survival curves were provided to calculate HRs for survival rates and their 95% confidence intervals (CIs). The eliminated criteria were as follows: (1) Repeated or overlapped articles; (2) Case-reports and reviews; (3) Inadequate original data of survival analysis. If the original article was not available for extracting or assessing data, the study was excluded. Two researchers (Lizhi Han and Bo Wang) screened all eligible studies elaborately, and a third researcher (Song Gong) was consulted to resolve any differences.

### Data Extraction

Two (Lizhi Han and Bo Wang) independently extracted related data and came to an agreement on all items. In order to obtain all qualified studies, the author, year of publication, tumor type, expression associated with poor prognosis, method of obtaining HRs, Newcastle–Ottawa Scale (NOS) score, and special information about the study population [such as number of patients (high/low), country of the population enrolled, follow up (month)], detection method, endpoints, survival analysis and cut-off value of all articles were collected. Overall survival (OS), disease-free survival (DFS), and progression-free survival (PFS) were all considered as endpoints. HR was extracted according to the previously proposed methodology to assess the impact of PANDAR expression on the prognosis of patients (26). We also inquired original data from the authors if they allowed.

### Quality Assessment

Quality of all included studies was evaluated independently by two researchers(Lizhi Han and Bo Wang) using the Newcastle-Ottawa Scale (27). There were totally three categories within the scale including selection, comparability, and outcome, with a full-mark of nine. Studies were identified as high-quality in methodology with at least six scores.

### Statistical Analysis

Stata Software 14.0 (Stata, College Station, TX) was used for quantitative calculation. For evaluating the prognostic value of PANDAR expression in different types of malignancies, pooled HRs (high/low) together with their related 95% CIs were applied. For analyzing the correlation between PANDAR expression levels and clinicopathological parameters, pooled ORs (high/low) and their related 95% CIs were utilized. Cochran's Q and *I*^2^ statistics were used to assess the heterogeneity among the included studies (28). An *I*^2^ value larger than 50% or a *p*-value lower than 0.10 was regarded as statistically significant. An insignificant heterogeneity (*p* > 0.01, *I*^2^ < 50%) was adjusted by a fixed-effects model for analysis, otherwise, a random-effects model was selected. Subgroup analysis and meta-regression were conducted to explore the source of heterogeneity. In addition, we could acquire clinicopathological characteristics from the studies, and figured out the pooled ORs and performed heterogeneity tests in order to analyze the relationship between PANDAR expression levels with tumor stages, tumor size, lymph node metastasis, gender, age, depth of invasion, differentiation grade, and distant metastasis in different kinds of cancers. Moreover, in order to check the stability of pooled outcomes, a sensitivity analysis was conducted. Both Begg's test and Egger's test were used for assessing publication bias (29). The statistical significance within all comparisons was mathematically signified as *P* < 0.05.

## Results

### Characteristics of Studies

**Figure 1:**
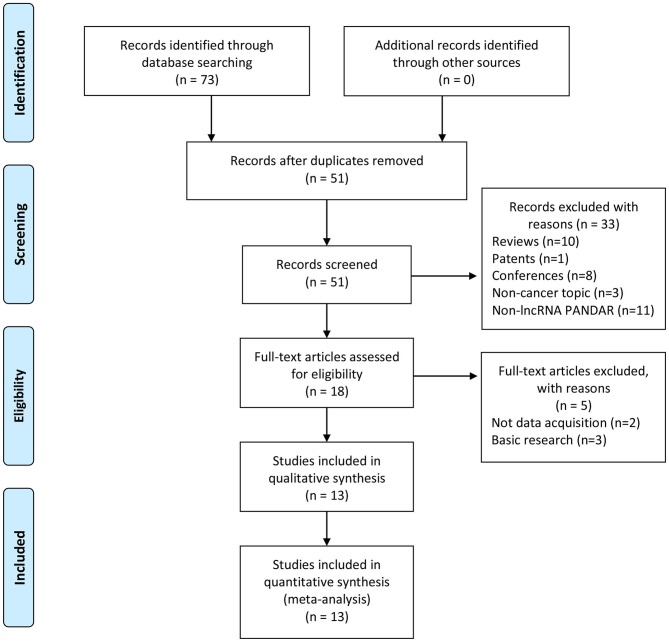
The selection flow chart of our systematic review.

Thirteen studies were selected from the 73 articles initially searched, which consists of 13 retrospective cohorts. Figure 1 shows the screening procedure and results in our study, according to PRISMA guideline (27). There were 1,465 patients involved in those studies, with 31 being the minimum sample size, and 482 being the largest. The accrual period was between 2015 and 2018. The enrolled studies were composed of ten types of cancers, including renal cell carcinoma (*n* = 2), colorectal cancer (*n* = 2), non-small cell lung cancer (*n* = 2), cervical squamous cell carcinoma (*n* = 1), gastric cancer (*n* = 1), cholangiocarcinoma (*n* = 1), hepatocellular carcinoma (*n* = 1), pancreatic ductal adenocarcinoma (*n* = 1), bladder cancer (*n* = 1), and oral squamous cell carcinoma (*n* = 1). OS, DFS, and PFS were reckoned as survival outcome, referring to 85% (11/13), 7% (1/13), and 7% (1/13), respectively, among these studies. In our study, EFS was derived from the combination of DFS and PFS, which was considered as a prognostic parameter. The lncRNA PANDAR expression levels were mainly measured using western blot (WB), real-time PCR (RT-PCR) and immunohistochemistry (IHC). Among these studies, the cut-off values were different due to various cut-off definitions. Further details about baseline features were recorded in Table 1.

**Table 1:** Characteristics of studies included in the meta-analysis.

**Study**	**Region**	**Tumor type**	**Detection method**	**Sample size** **(high/low)**	**Follow-up** **(month)**	**Endpoints**	**Expression associated with poor prognosis**	**Cut-off value**	**NOS score**	**Method**
Huang et al. (30)	China	Cervical squamous cell carcinoma	qRT-PCR	38/30 (68)	48	OS	High	High: fold change>4.7	6	1
Jiang et al. (31)	China	Pancreatic ductal adenocarcinoma	qRT-PCR WB	17/14 (31)	NA	OS	High	NA	6	2
Han et al. (21)	China	Non-small cell lung cancer	qRT-PCR WB IHC	70/70 (140)	60	OS	Low	Mean	6	1
Lu et al. (32)	China	Colorectal cancer	qRT-PCR WB	62/62 (124)	60	OS	High	Median	6	1
Ma et al. (17)	China	Gastric cancer	qRT-PCR	73/27 (100)	36	OS, DFS	High	NA	7	1
Peng and Fan (19)	China	Hepatocellular carcinoma	qRT-PCR	326/156 (482)	60	OS, TTR	High	NA	7	2
Li et al. (17)	China	Colorectal cancer	qRT-PCR	51/51 (102)	60	OS	High	Median	7	1
Xu et al. (20)	China	Renal cell carcinoma	qRT-PCR WB	34/28 (62)	40	OS	High	Median	6	1
Xu et al. (18)	China	Cholangiocarcinoma	qRT-PCR WB	40/27 (67)	60	OS	High	NA	7	1
Zhan et al. (33)	China	Bladder cancer	qRT-PCR	37/18 (55)	NA	OS	High	Mean	6	2
Jin (34)	China	Renal cell carcinoma	qRT-PCR	22/26 (48)	60	OS	High	NA	7	2
Huang et al. (35)	China	Oral squamous cell carcinoma	qRT-PCR	47/45 (92)	60	OS	High	NA	7	1
Nie et al. (36)	China	Non-small cell lung cancer	qRT-PCR	27/67 (94)	48	OS, PFS	Low	NA	6	2

*Method: 1 denoted as obtaining HRs directly from publications; 2 denoted as HRs calculated from the total number of events, corresponding p-value and data from Kaplan–Meier curves; IHC, immunohistochemistry; PCR, polymerase chain reaction; WB, western blot; OS, overall survival; TTR, time to recurrence; DFS, disease-free survival; NOS, Newcastle–Ottawa Scale; NA, not available*.

### Methodological Assessment

The majority of included trials were graded as high-quality in methodology, including six 7-score studies and seven 6-score studies. The detailed scores of each study by the Newcastle-Ottawa Scale were concluded in Supplementary Material 2.

### Association Between lncRNA PANDAR Expression Levels With OS of Cancer Patients

**Figure 2:**
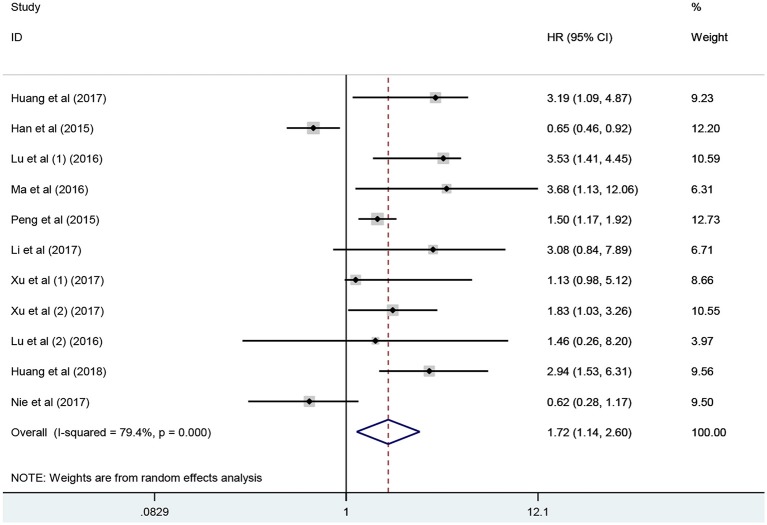
Meta-analysis of the pooled HR of OS for malignancy patients.

**Figure 3:**
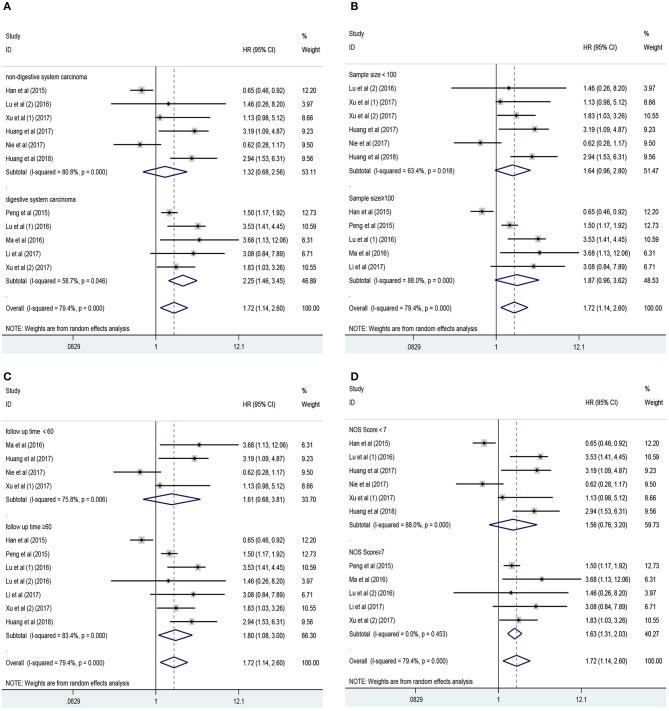
Results of subgroup analysis of pooled HR of OS for malignancy patients. **(A)** Subgroup analysis stratified by type of cancer. **(B)** Subgroup analysis stratified by sample size. **(C)** Subgroup analysis stratified by follow-up time. **(D)** Subgroup analysis stratified by NOS score.

Eleven studies consisting of 1,379 cancer patients reported the association between aberrant expression levels of lncRNA PANDAR with OS. The pooled HR was calculated using the random-effect model. Through detailed calculation, the pooled HR for OS was 1.72 (95%CI 1.14–2.60, *p* = 0.009), indicating the significant relationship between the high expression level of PANDAR with poor OS in malignancy patients (Figure 2). To investigate the source of significant heterogeneity among these studies (*I*^2^ = 79.4%, *p* < 0.001), subgroup analysis was further performed according to the following factors: type of cancer (non-digestive system or digestive system carcinoma), follow-up time (more than 60 or fewer than 60 months), sample size (more than 100 or fewer than 100), and paper quality (NOS scores ≥ 7 or < 7) (Figures 3A–D). The result of subgroup analysis demonstrated that the relationship between increased PANDAR expression levels with poor OS of cancer patients was still significant in all above factors apart from the subgroup of studies for non-digestive system carcinoma (HR 1.32, 95% CI 0.68–2.56, *p* = 0.419), follow-up time fewer than 60 months (HR 1.61, 95% CI 0.68–3.81, *p* = 0.275), and NOS scores <7 (HR 1.56, 95% CI 0.76–3.20, *p* = 0.230) (Table 2). In order to further explore the sources of heterogeneity, we conducted meta-regression through the covariates which also consisted of the above factors. However, the results that *p* values < 0.05 were not observed in the above covariates through meta-regression. This suggests that all above-mentioned factors were not the sources of heterogeneity (Table 2). In addition, we performed Cox multivariate analysis in nine studies including nine cohorts, finding that elevated expression level of PANDAR was an independent prognostic factor for OS in these cancer patients (HR 1.94, 95% CI 1.25–3.02, *p* = 0.003).

**Table 2:** Subgroup analysis of pooled HRs for OS in cancer patients with abnormal expression level of lncRNA PANDAR.

**Subgroup analysis**	**No. of cohorts**	**Pooled OR Random**	**Meta regression (*p* value)**	**Heterogeneity**	
				***I*^**2**^(%)**	***p* value**
Type of cancer			0.167		
Non-digestive system carcinoma	6	1.32 [0.68–2.56]	–	80.8	0.000
Digestive system carcinoma	5	2.25 [1.46–3.45]	–	58.7	0.046
Sample size			0.791		
≥100	5	1.87 [0.96–3.62]	–	88.0	0.000
<100	6	1.64 [0.96–2.80]	–	63.4	0.018
NOS scores			0.534		
≥7	5	1.63 [1.31–2.03]	–	00.0	0.453
<7	6	1.56 [0.76–3.20]	–	88.0	0.000
Follow-up time			0.786		
<60	4	1.61 [0.68–3.81]	–	75.8	0.006
≥60	7	1.80 [1.08–3.00]	–	83.4	0.000

### Association Between lncRNA PANDAR Expression Levels With EFS of Cancer Patients

**Figure 4:**
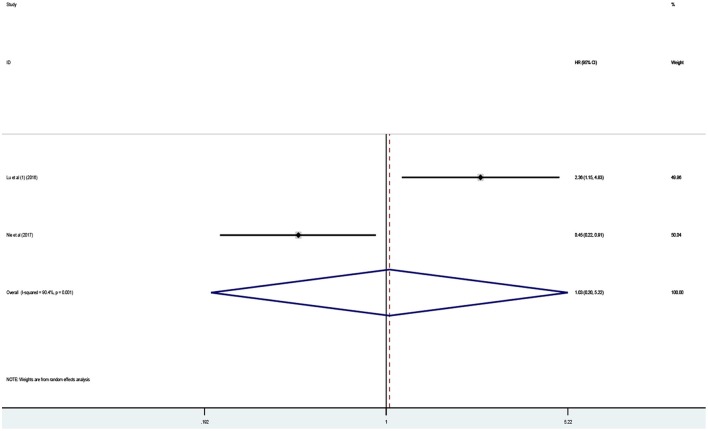
Meta-analysis of the pooled HR of EFS for malignancy patients.

Two studies in total, involving 194 patients, reported the effect of abnormally expressed PANDAR on DFS or PFS in cancer patients. In this current meta-analysis, DFS and PFS were defined as EFS. However, the outcome revealed no difference in predicting event-free survival (EFS) between the high and low expression of PANDAR (HR 1.03, 95% CI 0.20–5.22, *p* = 0.972) (Figure 4). Take the limited number of included studies into consideration, the subgroup analysis to explore the sources of heterogeneity was not performed.

### Association Between lncRNA PANDAR Expression Levels With OS of Certain Types of Cancers

**Figure 5:**
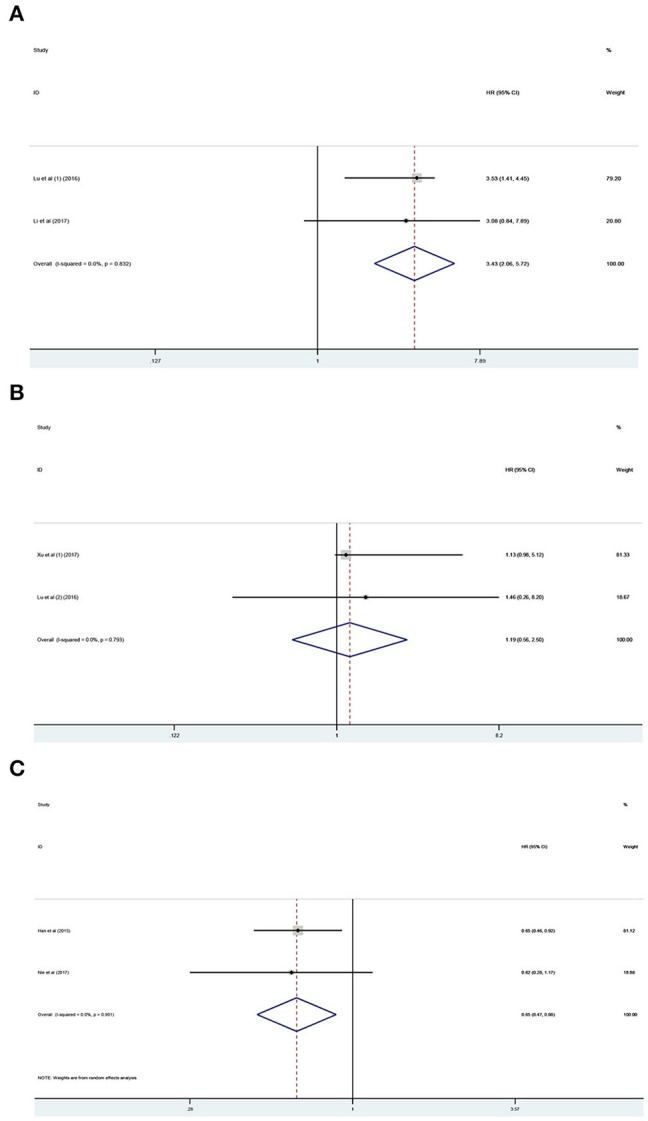
Meta-analysis of the pooled HR of OS for colorectal cancer **(A)**, renal cell carcinoma **(B)**, and non-small cell lung cancer **(C)**.

The prognostic value of PANDAR in various cancers was further evaluated. According to the results of systemic analysis, high PANDAR expression was related to reduced OS in colorectal cancer (HR 3.43; 95% CI 2.06–5.72, *p* < 0.001) (Figure 5A) and low expression level of PANDAR was significantly associated with poor OS in non-small cell lung cancer (pooled HR 0.65, 95%CI 0.45–0.88, *p* = 0.006) (Figure 5B). However, no significant association was noticed between the expression level of PANDAR and OS of patients suffering from renal cell carcinoma (HR 1.19; 95% CI 0.56–2.50, *p* = 0.655) (Figure 5C).

### Association Between lncRNA PANDAR Expression Levels With Clinicopathological Characteristics of Cancer Patients

**Figure 6:**
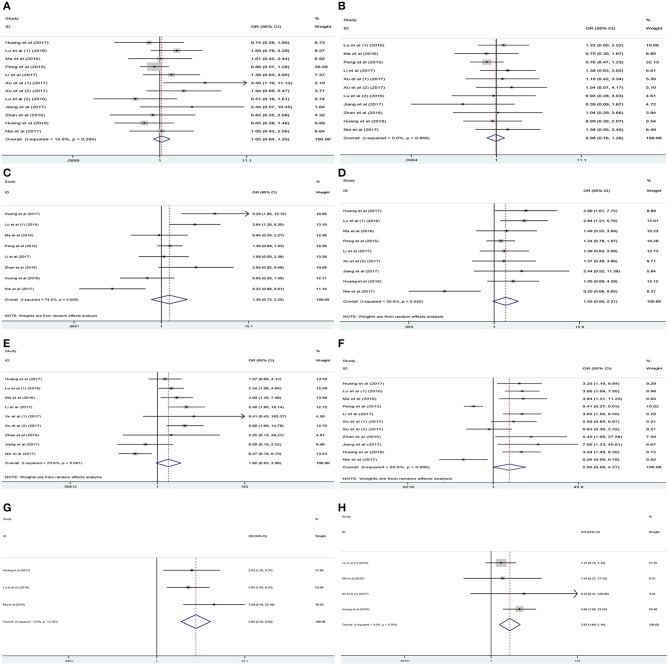
Association between PANDAR expression levels with clinicopathological characteristics of cancer patients. **(A)** Age, **(B)** gender, **(C)** tumor size, **(D)** differentiation grade, **(E)** lymph node metastasis, **(F)** tumor stage, **(G)** depth of invasion, **(H)** distant metastasis.

Analysis of the association between the expression levels of PANDAR and clinicopathological characteristics of cancer patients was illustrated in Table 3. The results of meta-analysis indicated that higher PANDAR expression levels were significantly associated with advanced tumor stage (OR = 2.05, 95% CI 0.99–4.27, *p* = 0.045) (Figure 6F), deeper depth of invasion (OR = 3.95, 95% CI 2.35–6.63, *p* < 0.001) (Figure 6G), more lymph node metastasis (OR = 1.92, 95% CI 0.93–3.98, *p* = 0.049) (Figure 6E), and farther distant metastasis (OR = 2.87, 95% CI 1.60–5.16, *p* < 0.001) (Figure 6H). However, no evidential relation was observed between elevated expression level of PANDAR with the older age (OR = 1.05, 95% CI 0.84–1.33, *p* = 0.649) (Figure 6A), gender (OR = 0.98, 95% CI 0.76–1.26, *p* = 0.879) (Figure 6B), larger tumor size (OR = 1.28, 95% CI 0.73–2.25, *p* = 0.386) (Figure 6C), and worse differentiation grade (OR = 1.45, 95% CI 0.95–2.21, *p* = 0.082) (Figure 6D).

**Table 3:** Association between lncRNA PANDAR and clinicopathological characteristics of cancer patients.

**Clinicopathological parameters**	**Studies (*n*)**	**Patients (*n*)**	**OR (95% CI)**	***P* value**	**Heterogeneity**		
					***I*^**2**^%**	***p***	**Model**
Age (>60 vs. ≤ 60 years)	12	1,330	1.05 (0.84–1.33)	0.649	15.8	0.289	Fixed
Gender (male vs. female)	11	1,207	0.98 (0.76–1.26)	0.879	0.00	0.856	Fixed
Tumor size (large vs. small)	8	1,117	1.28 (0.73–2.25)	0.386	74.5	0.000	Random
Differentiation grade (poorly and moderately VS well)	9	1,161	1.45 (0.95–2.21)	0.082	50.6	0.04	Random
Lymph node metastasis (yes vs. no)	9	702	1.92 (0.93–3.98)	0.049	70.0	0.001	Random
Tumor stage (III–IV vs. I–II)	11	1,248	2.05 (0.99–4.27)	0.045	85.6	0.000	Random
Depth of invasion	3	292	3.95 (2.35–6.63)	<0.001	0.00	0.381	Fixed
Distant metastasis	4	378	2.87 (1.60–5.16)	<0.001	0.00	0.393	Fixed

### Sensitivity Analysis

We performed the sensitivity analysis to evaluate the impacts of independent study on the overall outcomes. For OS, our sensitivity analysis revealed that results from Han et al. and Lu et al. had significant impacts on the outcomes, suggesting that these two studies were likely to be the main source of heterogeneity. However, after excluding single study one after another, the pooled HRs and 95% CIs list demonstrated the robustness of our results, with all pooled HRs and 95% CIs above the null hypothesis of 1.

### Publication Bias

**Figure 7:**
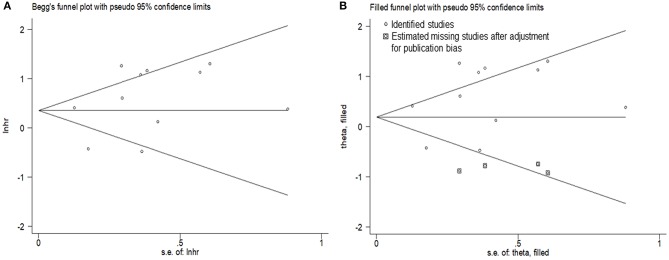
Begg's test **(A)** and trim and fill method funnel plot **(B)** for overall survival.

Visual inspection of the Begg funnel plot revealed asymmetry (Figure 7A). This raises the possibility of publication bias, although the Begg test was not statistically significant (*z* = 1.15; *P* = 0.28). As a result, we used the trim and fill method which conservatively imputes hypothetical negative unpublished studies to mirror the positive studies that cause funnel plot asymmetry. The imputed studies produce a symmetrical funnel plot (Figure 7B). The pooled analysis incorporating the hypothetical studies continued to show a statistically significant association between the high expression level of PANDAR with poor OS in malignancy patients (HR = 1.21, 95% CI 1.04–1.41, *p* = 0.012).

## Discussion

Cancer morbidity and mortality are rapidly growing around the world, and cancer is expected to be the leading cause of death and the most important obstacle for increasing life expectancy in every country of the world in this century (3). Although unable to translate into proteins, ncRNAs especially lncRNAs, are important in regulating growth, development, differentiation, gene expression and chromatin dynamics (37, 38). In the past few years, Next-Generation Sequencing(NGS) has shown that thousands of lncRNAs are abnormally expressed or mutated in various types of cancers (39). LncRNAs aberrantly expressed and mutated are closely associated with tumorigenesis, metastasis, and tumor stage (40–42). Due to its expression in certain types of cancers and its detection in circulating blood and/or urine, lncRNAs are a new kind of promising biomarkers and therapeutic targets for treating cancer with better diagnostic and prognostic value (43–45). Several cancer-associated lncRNAs which have been identified so far, are likely to be utilized as novel indicators for predicting tumor prognosis or as promising therapeutic targets for different types of cancers (46–51).

Recently, it has been found that PANDAR, as a novel tumor-associated lncRNA, exhibits abnormal expression in several cancers including gastric cancer (GC), colorectal cancer (CRC), renal cell carcinoma (RCC), bladder cancer (BC), hepatocellular carcinoma (HCC), cholangiocarcinoma (CCA), non-small cell lung cancer (NSCLC), and other cancers (17–21, 32, 33, 52, 53). However, inconsistent outcomes associated with PANDAR expression levels were found among several types of cancers including up-regulation in gastric cancer, hepatocellular carcinoma, colorectal cancer, thyroid cancer, osteosarcoma, breast cancer, clear cell renal cell carcinoma, and bladder cancer, whereas down-regulation in non-small cell lung cancer (16). As a kind of lncRNAs, PANDAR's expression level and its function can be variable in different types of cells, developmental states, and diseases, as a result of various interaction mechanisms and participating partners (54–58).

Due to the fact that the function of PANDAR in different cancers is still controversial and remains to be clarified, we conducted this meta-analysis to investigate the clinicopathological significance and prognostic value of abnormal PANDAR expression in patients suffering from cancer. Thirteen independent studies consisting of data from a total of 1,465 patients were systematically analyzed. Our results indicated that the high expression level of PANDAR was associated with poor OS significantly in cancer patients. Furthermore, we performed meta-regression and subgroup analysis to explore the sources of heterogeneity on account of the obvious heterogeneity across the studies. The outcomes of subgroup analysis showed that the prognostic significance of PANDAR was altered by the type of cancer (non-digestive system carcinoma), follow-up time (<60 months) and paper quality (NOS scores <7). Therefore, we could conclude that the difference in the specific type of cancer, follow-up time and paper quality was likely to be the source of heterogeneity. However, meta-regression analysis failed to identify the source of the obvious heterogeneity in above covariates. Moreover, we found that PANDAR was an independent prognostic factor of OS in cancer patients when combined with HRs from Cox multivariate analysis. PFS and DFS, defined as EFS in this meta-analysis, are important parameters that reflect tumor progression. The prognostic significance of PANDAR in EFS was also assessed in 2 studies including 194 patients. Our results showed that the association between the reduced expression level of PANDAR with poor EFS in cancer patients was not significant.

Furthermore, the prognostic value of PANDAR in certain types of cancer was evaluated. The results indicated that increased PANDAR expression was closely related to reduced OS in colorectal cancer, whereas reduced PANDAR expression was associated with decreased OS of non-small cell lung cancer. However, no significant relationship between the expression level of PANDAR and the OS of renal cell carcinoma was observed. Therefore, we could conclude that the expression level of PANDAR might play different roles in predicting OS in several kinds of cancers.

With regard to the clinicopathological characteristics, our analysis demonstrated that elevated expression level of PANDAR was associated significantly with advanced tumor stage, deeper depth of invasion, more lymph node metastasis and farther distant metastasis. However, no significant relation was found between PANDAR expression levels with older age, gender, larger tumor size and worse differentiation grade of cancer patients.

In spite of the inspiring results, there are still several disadvantages in this quantitative meta-analysis. Firstly, although the random-effect model and subgroup analysis were used, the heterogeneity among the studies was still not completely eliminated, which might bring about the bias of the results to some extent. Secondly, the cut-off value for abnormal expression of PANDAR was varied among the included studies, which could result in the bias of the outcomes. Thirdly, although the baseline figures were comparable, our summary analysis depended too much on the strength of including cohort above all, which might partially result in selection bias. Finally, some HRs could not be obtained from the included studies directly, which could make calculations from survival curves inaccurate.

## Conclusions

From our study, we can conclude that elevated expression level of PANDAR may be a poor prognostic biomarker for OS. However, in this meta-analysis, there was no significant association between the expression level of PANDAR with EFS. In addition, our review revealed that elevated expression level of PANDAR was associated with decreased OS in colorectal cancer, whereas the reduced PANDAR expression level was significantly related to poor OS in non-small cell lung cancer. However, no significant relation was found between PANDAR expression level with OS of patients suffering from renal cell carcinoma. Moreover, PANDAR expression level was related to clinicopathological characteristics including TNM stage, depth of invasion, lymph node metastasis, and distant metastasis. In order to explore the more important role of PANDAR in human cancer, more relevant researches will be needed in the future.

## Data Availability Statement

The datasets during and/or analyzed during the current study available from the corresponding author on reasonable request.

## Author Contributions

YF and WX conceived and designed this study. LH, BW, and ZW were responsible for the collection, extraction, and analysis of the data. LH was responsible for writing the paper. RW, SG, and GC performed the quality evaluation and completed data analysis. DT polished the English language. All authors and participants reviewed the paper and reached an agreement to approve the final manuscript.

### Conflict of Interest

The authors declare that the research was conducted in the absence of any commercial or financial relationships that could be construed as a potential conflict of interest.

## Abbreviations

GC: gastric cancer
CRC: colorectal cancer
RCC: renal cell carcinoma
BC: bladder cancer
HCC: hepatocellular carcinoma
CCA: cholangiocarcinoma
NSCLC: non-small cell lung cancer
HR: hazard ratios
OR: Odds ratios
CI: confidence intervals
OS: overall survival
PFS: progression-free survival
DFS: disease-free survival
WB: western blot
PCR: polymerase chain reaction
IHC: immunohistochemistry
NOS: Newcastle–Ottawa Scale.

## References

[1] Lichtenstein AV Strategies of the war on cancer: to kill or to neutralize? Front Oncol. (2018) 8:667 10.3389/fonc.2018.00667 30687641 PMC6335948

[2] Siegel RL Miller KD Jemal A Cancer statistics, 2018. CA Cancer J Clin. (2018) 68:7–30. 10.3322/caac.21442 29313949

[3] Bray F Ferlay J Soerjomataram I Siegel RL Torre LA Jemal A. Global cancer statistics 2018: GLOBOCAN estimates of incidence and mortality worldwide for 36 cancers in 185 countries. CA Cancer J Clin. (2018) 68:394–424. 10.3322/caac.21492 30207593

[4] Ferlay J Soerjomataram I Dikshit R Eser S Mathers C Rebelo M. Cancer incidence and mortality worldwide: sources, methods and major patterns in GLOBOCAN 2012. Int J Cancer. (2015) 136:E359–86. 10.1002/ijc.29210 25220842

[5] Shi XS Li J Yang RH Zhao GR Zhou HP Zeng WX. Correlation of increased MALAT1 expression with pathological features and prognosis in cancer patients: a meta-analysis. Genet Mol Res. (2015) 14:18808–19. 10.4238/2015.December.28.30 26782531

[6] Frith MC Bailey TL Kasukawa T Mignone F Kummerfeld SK Madera M. Discrimination of non-protein-coding transcripts from protein-coding mRNA. RNA Biol. (2006) 3:40–8. 10.4161/rna.3.1.2789 17114936

[7] Mercer TR Mattick JS. Structure and function of long noncoding RNAs in epigenetic regulation. Nat Struct Mol Biol. (2013) 20:300–7. 10.1038/nsmb.2480 23463315

[8] Sanchez Calle A Kawamura Y Yamamoto Y Takeshita F Ochiya T. Emerging roles of long non-coding RNA in cancer. Cancer Sci. (2018) 109:2093–100. 10.1111/cas.13642 29774630 PMC6029823

[9] Chen D Lu T Tan J Li H Wang Q Wei L. Long non-coding RNAs as communicators and mediators between the tumor microenvironment and cancer cells. Front Oncol. (2019) 9:739. 10.3389/fonc.2019.00739 31448238 PMC6691164

[10] Bhan A Soleimani M Mandal SS. Long noncoding RNA and cancer: a new paradigm. Cancer Res. (2017) 77:3965–81. 10.1158/0008-5472.CAN-16-2634 28701486 PMC8330958

[11] Wang KC Chang HY. Molecular mechanisms of long noncoding RNAs. Mol Cell. (2011) 43:904–14. 10.1016/j.molcel.2011.08.018 21925379 PMC3199020

[12] Yoon JH Abdelmohsen K Srikantan S Yang X Martindale JL De S. LincRNA-p21 suppresses target mRNA translation. Mol Cell. (2012) 47:648–55. 10.1016/j.molcel.2012.06.027 22841487 PMC3509343

[13] Kung JT Colognori D Lee JT. Long noncoding RNAs: past, present, and future. Genetics. (2013) 193:651–69. 10.1534/genetics.112.146704 23463798 PMC3583990

[14] Hung T Wang Y Lin MF Koegel AK Kotake Y Grant GD. Extensive and coordinated transcription of noncoding RNAs within cell-cycle promoters. Nat Genet. (2011) 43:621–9. 10.1038/ng.848 21642992 PMC3652667

[15] Sang Y Tang J Li S Li L Tang X Cheng C. LncRNA PANDAR regulates the G1/S transition of breast cancer cells by suppressing p16(INK4A) expression. Sci Rep. (2016) 6:22366. 10.1038/srep22366 26927017 PMC4772134

[16] Li J Li Z Zheng W Li X Wang Z Cui Y. PANDAR: a pivotal cancer-related long non-coding RNA in human cancers. Mol Biosyst. (2017) 13:2195–201. 10.1039/C7MB00414A 28976505

[17] Ma P Xu T Huang M Shu Y. Increased expression of LncRNA PANDAR predicts a poor prognosis in gastric cancer. Biomed Pharmacother. (2016) 78:172–6. 10.1016/j.biopha.2016.01.025 26898439

[18] Xu Y Jiang X Cui Y. Upregulated long noncoding RNA PANDAR predicts an unfavorable prognosis and promotes tumorigenesis in cholangiocarcinoma. Onco Targets Ther. (2017) 10:2873–83. 10.2147/OTT.S137044 28652769 PMC5476724

[19] Peng W Fan H. Long non-coding RNA PANDAR correlates with poor prognosis and promotes tumorigenesis in hepatocellular carcinoma. Biomed Pharmacother. (2015) 72:113–8. 10.1016/j.biopha.2015.04.014 26054684

[20] Xu Y Tong Y Zhu J Lei Z Wan L Zhu X. An increase in long non-coding RNA PANDAR is associated with poor prognosis in clear cell renal cell carcinoma. BMC Cancer. (2017) 17:373. 10.1186/s12885-017-3339-9 28545465 PMC5445460

[21] Han L Zhang EB Yin DD Kong R Xu TP Chen WM. Low expression of long noncoding RNA PANDAR predicts a poor prognosis of non-small cell lung cancer and affects cell apoptosis by regulating Bcl-2. Cell Death Dis. (2015) 6:e1665. 10.1038/cddis.2015.30 25719249 PMC4669812

[22] Cabili MN Trapnell C Goff L Koziol M Tazon-Vega B Regev A. Integrative annotation of human large intergenic noncoding RNAs reveals global properties and specific subclasses. Genes Dev. (2011) 25:1915–27. 10.1101/gad.17446611 21890647 PMC3185964

[23] Derrien T Johnson R Bussotti G Tanzer A Djebali S Tilgner H. The GENCODE v7 catalog of human long noncoding RNAs: analysis of their gene structure, evolution, and expression. Genome Res. (2012) 22:1775–89. 10.1101/gr.132159.111 22955988 PMC3431493

[24] Liberati A Altman DG Tetzlaff J Mulrow C Gotzsche PC Ioannidis JP. The PRISMA statement for reporting systematic reviews and meta-analyses of studies that evaluate healthcare interventions: explanation and elaboration. BMJ. (2009) 339:b2700. 10.1136/bmj.b2700 19622552 PMC2714672

[25] Ekpu VU Brown AK. The economic impact of smoking and of reducing smoking prevalence: review of evidence. Tob Use Insights. (2015) 8:1–35. 10.4137/TUI.S15628 26242225 PMC4502793

[26] Parmar MK Torri V Stewart L. Extracting summary statistics to perform meta-analyses of the published literature for survival endpoints. Stat Med. (1998) 17:2815–34. 10.1002/(SICI)1097-0258(19981230)17:24<2815::AID-SIM110>3.0.CO;2-8 9921604

[27] Zeng X Zhang Y Kwong JS Zhang C Li S Sun F. The methodological quality assessment tools for preclinical and clinical studies, systematic review and meta-analysis, and clinical practice guideline: a systematic review. J Evid Based Med. (2015) 8:2–10. 10.1111/jebm.12141 25594108

[28] Wijarnpreecha K Thongprayoon C Panjawatanan P Ungprasert P. Hepatitis C virus infection and risk of osteoporotic fracture: a systematic review and meta-analysis. J Evid Based Med. (2018) 11:20–5. 10.1111/jebm.12286 29322660

[29] Boonpheng B Thongprayoon C Cheungpasitporn W. The comparison of risk of stroke in patients with peritoneal dialysis and hemodialysis: a systematic review and meta-analysis. J Evid Based Med. (2018) 11:158–68. 10.1111/jebm.12315 30070027

[30] Huang HW Xie H Ma X Zhao F Gao Y. Upregulation of LncRNA PANDAR predicts poor prognosis and promotes cell proliferation in cervical cancer. Eur Rev Med Pharmacol Sci. (2017) 21:4529–35. 29131264

[31] Jiang Y Feng E Sun L Jin W You Y Yao Y. An increased expression of long non-coding RNA PANDAR promotes cell proliferation and inhibits cell apoptosis in pancreatic ductal adenocarcinoma. Biomed Pharmacother. (2017) 95:685–91. 10.1016/j.biopha.2017.08.124 28886528

[32] Lu M Liu Z Li B Wang G Li D Zhu Y. The high expression of long non-coding RNA PANDAR indicates a poor prognosis for colorectal cancer and promotes metastasis by EMT pathway. J Cancer Res Clin Oncol. (2017) 143:71–81. 10.1007/s00432-016-2252-y 27629879 PMC11818975

[33] Zhan Y Lin J Liu Y Chen M Chen X Zhuang C. Up-regulation of long non-coding RNA PANDAR is associated with poor prognosis and promotes tumorigenesis in bladder cancer. J Exp Clin Cancer Res. (2016) 35:83. 10.1186/s13046-016-0354-7 27206339 PMC4873988

[34] Jin L Expression and clinical significance of long non-coding RNA PANDAR in renal cell carcinoma. Acta Univ Med Anhui. (2016) 51 10.19405/j.cnki.issn1000-1492.2016.09.024

[35] Huang Z Sang T Zheng Y Wu J Long non-coding RNA PANDAR overexpression serves as a poor prognostic biomarker in oral squamous cell carcinoma. Int J Clin Exp Pathol. (2018) 11:2728–34. PMC6958275 31938389

[36] Nie J Zhou B Zhang Y The expression and clinical significance of PANDAR in non-small cell lung cancer. China Oncol. (2017) 27:569–74. 10.19401/j.cnki.1007-3639.2017.07.008

[37] Guttman M Amit I Garber M French C Lin MF Feldser D. Chromatin signature reveals over a thousand highly conserved large non-coding RNAs in mammals. Nature. (2009) 458:223–7. 10.1038/nature07672 19182780 PMC2754849

[38] Bhan A Mandal SS. LncRNA HOTAIR: A master regulator of chromatin dynamics and cancer. Biochim Biophys Acta. (2015) 1856:151–64. 10.1016/j.bbcan.2015.07.001 26208723 PMC4544839

[39] Bhan A Mandal SS. Long noncoding RNAs: emerging stars in gene regulation, epigenetics and human disease. ChemMedChem. (2014) 9:1932–56. 10.1002/cmdc.201300534 24677606

[40] Kornfeld JW Bruning JC. Regulation of metabolism by long, non-coding RNAs. Front Genet. (2014) 5:57. 10.3389/fgene.2014.00057 24723937 PMC3971185

[41] Vitiello M Tuccoli A Poliseno L Long non-coding RNAs in cancer: implications for personalized therapy. Cell Oncol (Dordr). (2015) 38:17–28. 10.1007/s13402-014-0180-x 25113790 PMC13004270

[42] Bartonicek N Maag JL Dinger ME. Long noncoding RNAs in cancer: mechanisms of action and technological advancements. Mol Cancer. (2016) 15:43. 10.1186/s12943-016-0530-6 27233618 PMC4884374

[43] Brunner AL Beck AH Edris B Sweeney RT Zhu SX Li R. Transcriptional profiling of long non-coding RNAs and novel transcribed regions across a diverse panel of archived human cancers. Genome Biol. (2012) 13:R75. 10.1186/gb-2012-13-8-r75 22929540 PMC4053743

[44] Yan X Hu Z Feng Y Hu X Yuan J Zhao SD. Comprehensive genomic characterization of long non-coding RNAs across human cancers. Cancer Cell. (2015) 28:529–40. 10.1016/j.ccell.2015.09.006 26461095 PMC4777353

[45] Shi T Gao G Cao Y. Long noncoding RNAs as novel biomarkers have a promising future in cancer diagnostics. Dis Markers. (2016) 2016:9085195. 10.1155/2016/9085195 27143813 PMC4842029

[46] Zhang S Chen S Yang G Gu F Li M Zhong B. Long noncoding RNA HOTAIR as an independent prognostic marker in cancer: a meta-analysis. PLoS ONE. (2014) 9:e105538. 10.1371/journal.pone.0105538 25157956 PMC4144893

[47] Zhu L Liu J Ma S Zhang S. Long noncoding RNA MALAT-1 can predict metastasis and a poor prognosis: a meta-analysis. Pathol Oncol Res. (2015) 21:1259–64. 10.1007/s12253-015-9960-5 26159858

[48] Liu FT Pan H Xia GF Qiu C Zhu ZM. Prognostic and clinicopathological significance of long noncoding RNA H19 overexpression in human solid tumors: evidence from a meta-analysis. Oncotarget. (2016) 7:83177–86. 10.18632/oncotarget.13076 27825121 PMC5347760

[49] Serghiou S Kyriakopoulou A Ioannidis JP. Long noncoding RNAs as novel predictors of survival in human cancer: a systematic review and meta-analysis. Mol Cancer. (2016) 15:50. 10.1186/s12943-016-0535-1 27352941 PMC4924330

[50] Fan YH Fang H Ji CX Xie H Xiao B Zhu XG. Long noncoding RNA CCAT2 can predict metastasis and poor prognosis: a meta-analysis. Clin Chim Acta. (2017) 466:120–6. 10.1016/j.cca.2017.01.016 28089750

[51] Fan YH Ye MH Wu L Wu MJ Lu SG Zhu XG. BRAF-activated lncRNA predicts gastrointestinal cancer patient prognosis: a meta-analysis. Oncotarget. (2017) 8:6295–303. 10.18632/oncotarget.14061 28009984 PMC5351632

[52] Li X Wang F Sun Y Fan Q Cui G. Expression of long non-coding RNA PANDAR and its prognostic value in colorectal cancer patients. Int J Biol Markers. (2017) 32:e218–23. 10.5301/jbm.5000249 28106228

[53] Li Z Gao B Hao S Tian W Chen Y Wang L. Knockdown of lncRNA-PANDAR suppresses the proliferation, cell cycle and promotes apoptosis in thyroid cancer cells. EXCLI J. (2017) 16:354–62. 10.17179/excli2017-113 28507479 PMC5427478

[54] Wapinski O Chang HY. Long noncoding RNAs and human disease. Trends Cell Biol. (2011) 21:354–61. 10.1016/j.tcb.2011.04.001 21550244

[55] Guttman M Rinn JL. Modular regulatory principles of large non-coding RNAs. Nature. (2012) 482:339–46. 10.1038/nature10887 22337053 PMC4197003

[56] Rinn JL Chang HY. Genome regulation by long noncoding RNAs. Annu Rev Biochem. (2012) 81:145–66. 10.1146/annurev-biochem-051410-092902 22663078 PMC3858397

[57] Neguembor MV Jothi M Gabellini D. Long noncoding RNAs, emerging players in muscle differentiation and disease. Skelet Muscle. (2014) 4:8. 10.1186/2044-5040-4-8 24685002 PMC3973619

[58] Ward M McEwan C Mills JD Janitz M. Conservation and tissue-specific transcription patterns of long noncoding RNAs. J Hum Transcr. (2015) 1:2–9. 10.3109/23324015.2015.1077591 27335896 PMC4894084

