# Supplementary material for: Prognostic and Clinicopathological Significance of Long Non-coding RNA PANDAR Expression in Cancer Patients: A Meta-Analysis
Source: Front Oncol. 2019 Dec 3;9:1337. doi: 10.3389/fonc.2019.01337 (PMC6901660; doi:10.3389/fonc.2019.01337)
Supplement: Supplementary file 1 [file Table_1.DOCX]

**Supplementary Material 1** The detailed example of the full electronic search strategy for PubMed

(((((((((((((((((((Neoplasia[Title/Abstract]) OR Neoplasias[Title/Abstract]) OR Neoplasm[Title/Abstract]) OR Tumors[Title/Abstract]) OR Tumor[Title/Abstract]) OR Cancer[Title/Abstract]) OR Cancers[Title/Abstract]) OR Malignancy[Title/Abstract]) OR Malignancies[Title/Abstract]) OR Malignant Neoplasms[Title/Abstract]) OR Malignant Neoplasm[Title/Abstract]) OR Neoplasm, Malignant[Title/Abstract]) OR Neoplasms, Malignant[Title/Abstract]) OR Benign Neoplasms[Title/Abstract]) OR Neoplasms, Benign[Title/Abstract]) OR Benign Neoplasm[Title/Abstract]) OR Neoplasm, Benign[Title/Abstract]) OR "Neoplasms"[Mesh])) AND "long non-coding RNA PANDAR, human" [Supplementary Concept]
