# Supplementary material for: Prognostic and Clinicopathological Significance of Long Non-coding RNA PANDAR Expression in Cancer Patients: A Meta-Analysis
Source: Front Oncol. 2019 Dec 3;9:1337. doi: 10.3389/fonc.2019.01337 (PMC6901660; doi:10.3389/fonc.2019.01337)
Supplement: Supplementary file 2 [file Table_2.DOCX]

**Supplementary Material 2** Methodological assessment by Newcastle-Ottawa Scale

| Study | Selection | Comparability | Outcome | Total |
| --- | --- | --- | --- | --- |
| Huang 2017 | 3 | 1 | 2 | 6 |
| Jiang 2017 | 2 | 2 | 2 | 6 |
| Han 2015 | 3 | 1 | 2 | 6 |
| Lu 2016 | 3 | 1 | 2 | 6 |
| Ma 2016 | 3 | 2 | 2 | 7 |
| Peng 2015 | 3 | 2 | 2 | 7 |
| Li 2017 | 3 | 2 | 2 | 7 |
| Xu 2017 (1) | 2 | 2 | 2 | 6 |
| Xu 2017 (2) | 3 | 1 | 3 | 7 |
| Zhan 2016 | 3 | 1 | 2 | 6 |
| Jin 2016 | 3 | 2 | 2 | 7 |
| Huang 2018 | 2 | 2 | 3 | 7 |
| Nie 2017 | 3 | 1 | 2 | 6 |
